# Genome data mining of a novel *Stutzerimonas marianensis* strain LB-0542 isolated from pelagic *Sargassum* seaweed waste for plastic-degrading and plant growth-promoting traits

**DOI:** 10.1016/j.dib.2026.112454

**Published:** 2026-01-08

**Authors:** Bidyut R. Mohapatra, Linel S. Moralez, Kiya E. James

**Affiliations:** Department of Biological and Chemical Sciences, The University of the West Indies, Cave Hill Campus, Bridgetown BB11000, Barbados

**Keywords:** Annotation, Biocatalyst, Biomass-degrading enzyme, Carbohydrate-active enzyme, Culturomics, Macroalgae, Next-generation sequencing, Phylogenomics

## Abstract

This study reports the whole-genome sequence data and functional annotations of a novel *Stutzerimonas marianensis* strain LB-0542 isolated from the decomposing pelagic *Sargassum* biomass stranded on Long Beach, Barbados. The genomic DNA was sequenced with the Illumina NextSeq2000 platform. The genome assembly was performed with the SPAdes Genome Assembler (ver 3.15.5). The assembled genome has a size of 4520,813 bp, a coverage of 110X, a GC content of 63.2 %, a L_50_ of 2 and a N_50_ of 1079,143 bp. The genome consists of 12 contigs, 0 CRISPR, 3 rRNA, 56 tRNA and 4166 CDSs (coding sequences) with a coding ratio of 89.4 %. The genome annotation results for the COG (cluster of orthologous genes) and subsystem features indicate that the metabolism and the amino acids and derivatives are the most dominant categories, respectively. The analysis of the genome for the existence of Carbohydrate-Active Enzymes (CAZymes) identified 230 genes encoding four functional classes of CAZymes [glycoside hydrolases (75 genes), glycosyltransferases (95 genes), carbohydrate esterases (9 genes) and carbohydrate-binding modules (51 genes)]. The functional annotation of the genome for plastic degradation revealed the presence of 34 genes, which could catalyse the degradation process of 14 types of plastics, polyethylene glycol [PEG (29 %)], polylactic acid [PLA (11 %)], poly(3-hydroxybutyrate-co-3-hydroxyvalerate) [PHBV (9 %)], polyhydroxyalkanoates [PHA (9 %)], polyethylene [PE (6 %)], polycaprolactone [PCL (6 %)], polyethersulfone [PES (6 %)], polyethylene terephthalate [PET (6 %)], poly(butylene adipate-co-terephthalate [PBAT (3 %)], (polystyrene [PS (3 %)], polybutylene succinate [PBSA (3 %)], poly(3-hydroxyvalerate) [P3HV (3 %)], polyvinyl alcohol [PVA (3 %)] and natural rubber [NR (3 %)]. The genome mining for plant growth-promoting traits identified 3175 genes that are associated with the colonizing plant system (26 %), competitive exclusion (21 %), stress control (21 %), biofertilization (14 %), phytohormone and plant signal production (10 %), bioremediation (7 %) and plant immune response stimulation (1 %). These genome mining results are an indication of the biotechnological and ecological significance of the novel strain LB-0542 for sustainable biocatalytic processing of *Sargassum* and plastic-containing waste. The genome sequence data is available in DDBJ/EMBL/GenBank with the accession number BAAIAE000000000.

Specifications Table

**Table:** 

Subject	Biology
Specific subject area	Microbiology, Genomics, Biotechnology
Type of data	Raw and processed genome sequence (Figures and tables)
Data collection	The genomic DNA was sequenced with the Illumina NextSeq2000 platform and paired-end library preparation protocol. The genome was assembled with the SPAdes Genome Assembler (ver 3.15.5) and annotated using the DDBJ Fast Annotation and Submission Tool (DFAST). Functional annotations with reference to the clusters of orthologous genes (COG), subsystem features, carbohydrate-active enzymes, plastic degradation and plant growth-promoting traits were performed using the eggNOG mapper (ver. 4.1), Rapid Annotation System Technology (RAST), Protologger (ver 2.0), Plastic Biodegradation Database (PlasticDB) and Plant Growth-Promoting Traits Prediction (PGPT-Pred) tools, respectively.
Data source location	The strain LB-0542 used in this study was isolated from the decomposing pelagic *Sargassum* biomass stranded on Long Beach (13.06°N, 59.49°W), Barbados.
Data accessibility	Repository name: NCBI GenBank Data identification number for NCBI (accession number): BAAIAE000000000 Direct URL to NCBI data: https://www.ncbi.nlm.nih.gov/nuccore/BAAIAE000000000

## 1 Value of the Data

• The genomic data provides an insight into the evolutionary relationships and metabolic characteristics of the *Stutzerimonas* species.
• The genomic data reveals the industrial significance of *Stutzerimonas* species in sustainable biodegradation of plastic- and polysaccharide-containing waste.
• The genomic data is valuable in assessing the molecular mechanism involved in microbe-plant beneficial interactions.

## 2 Background

The deposition of millions of tons of pelagic *Sargassum* seaweed biomass off the coasts of the tropical and subtropical Atlantic Ocean is an environmental and public health concern [1]. The causal agents of seaweed blooming include the changes in global climate and land-use practices [2]. Recent studies reported the accumulation of microplastics in the tissues of the bloom-forming pelagic *Sargassum* species [3,4]. The retention of the microplastics in the tissues of the *Sargassum* species is attributable to the electrostatic interaction between microplastics and alginate (a major structural polysaccharide) and/or adherence/entanglement in the algal biomass [4]. In recent years, there has been significant industrial interest in the biorefining of beached *Sargassum* biomass leading to value-added products with applications in agriculture, food, energy and pharmaceuticals [5]. *Sargassum* biomass is rich in carbohydrates, minerals, proteins and vitamins [6]. Therefore, the decomposing *Sargassum* biomass (aka *Sargassum* waste) is a suitable ecosystem for harbouring a diverse group of microorganisms, which should catalyse the natural degradation process by producing an array of metabolites [[6], [7], [8]]. In order to evaluate the biotechnological significance of *Sargassum* waste associated microorganisms, it is essential to characterize their phylogeny and metabolic traits via integration of culturomics and whole-genome sequencing. This paper reports the genomic features, phylogenomics and functional annotations with respect to the carbohydrate-active enzymes, plastic degradation and plant-growth promoting traits of a novel *Stutzerimonas marianensis* strain LB-0542 isolated from the pelagic *Sargassum* waste.

## 3 Data Description

**Fig. 1:**
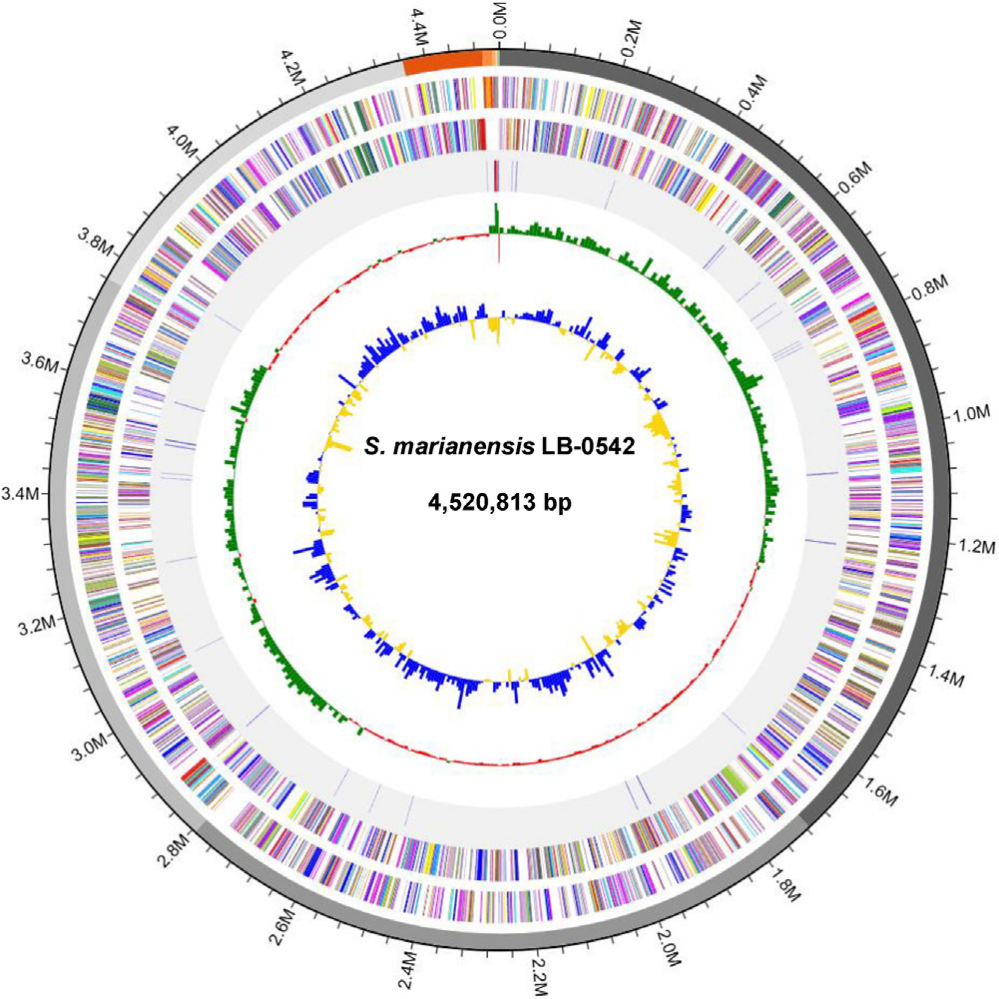
A genome map of the novel *S. marianensis* strain LB-0542. From the outermost to innermost, circle 1: 12 assembled contigs; circle 2: CDSs in forward strand; circle 3: CDSs in reverse strand. Each CDS is highlighted with a color according to its COG category (https://help.ezbiocloud.net/cog-colors/). Circle 4: rRNA and tRNA; circle 5: GC skew; circle 6: GC ratio. The color descriptions for the circles 4, 5 and 6 are available in https://help.ezbiocloud.net/genome-map/.

**Fig. 2:**
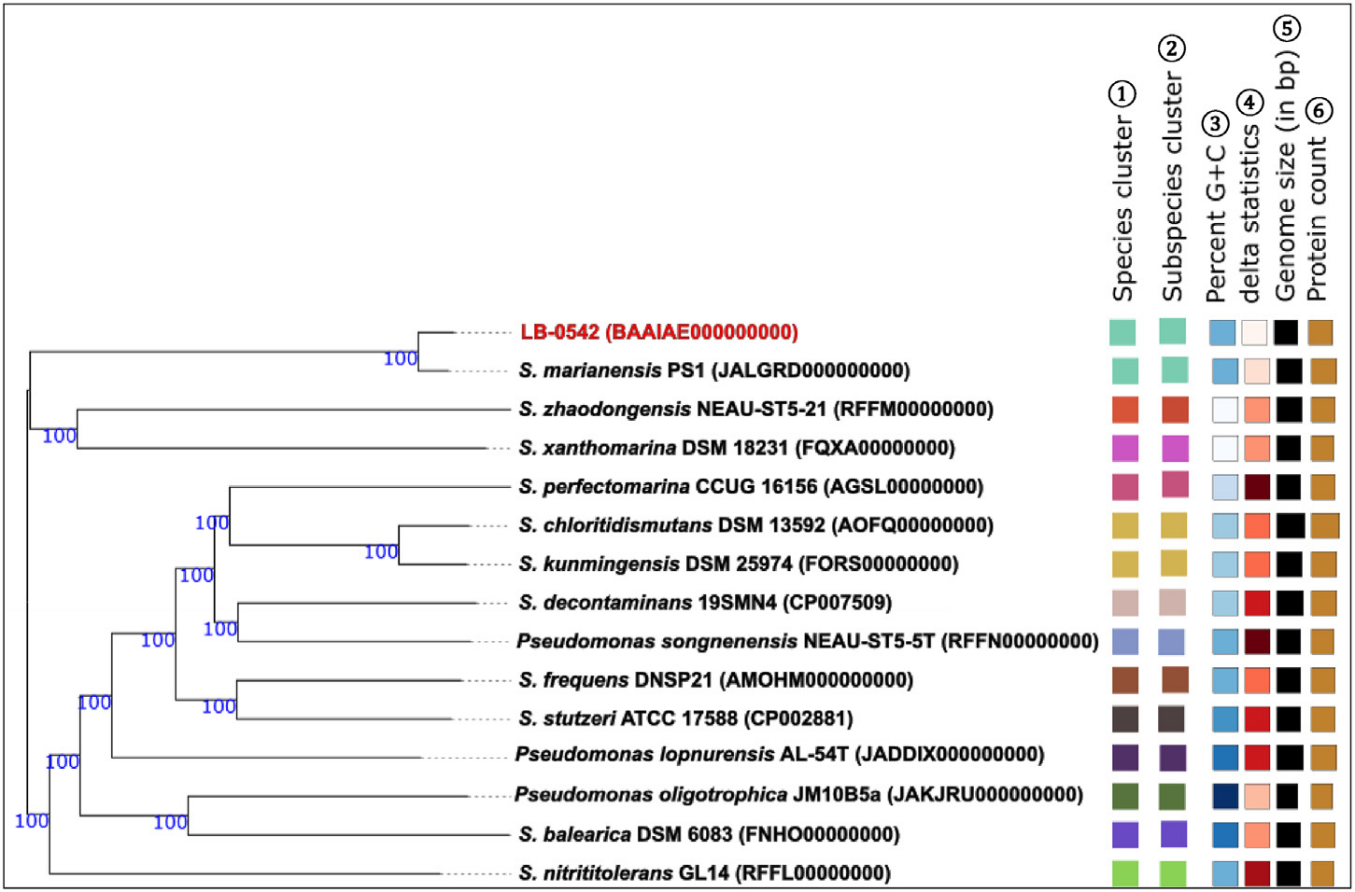
Genome-based evolutionary relatedness of the novel *S. marianensis* strain LB-0542. The tree was constructed using the FastME 2.1.6.1 program available in TYGS [20] and the Genome Blast Distance Phylogeny (GBDP) distances calculated from genome sequences. The branch lengths are scaled in terms of the GBDP distance formula *d_5_*. The GBDP pseudo-bootstrap support values >60 % from 100 replications (average branch support 100 %) are specified above the branches. The tree was rooted at the midpoint. Leaf labels are interpreted by affiliation to (1) dDDH species cluster (min 22.2–max 86.1), (2) dDDH subspecies cluster (min 22.2–max 86.1), (3) percent *G* + *C* [min (light blue) 59.6–max (dark blue) 67.2], (4) δ value [min (light brown) 0.118–max (dark brown) 0.205], (5) genome size (min 3.97 Mbp–max 5.05 Mbp) and (6) protein count (min 3688–max 4767). The DDBJ/EMBL/GenBank accession numbers are specified in parentheses.

The genome size of the strain LB-0542 was recorded as 4520,813 bp with a coverage of 110X, a GC content of 63.2 % and a gap ratio of 0 %. The assembled genome generated 12 contigs with the L_50_ value of 2, the N_50_ of 1079,143 bp and the longest contig of 1715,002 bp. The quality check of the LB-0542 assembled genome via the CheckM program in the DDBJ Fast Annotation and Submission Tool (DFAST) [9] revealed 99.4 % completeness and 0.44 % contamination. Additional quality checking using the ContEst16S (Contamination Estimator by 16S) [10] indicated no contamination of the genome. The annotation via the DFAST indicates that the genome consisted of 3 rRNA, 56 tRNA, 0 CRISPR, 4166 CDSs (coding sequences) and a coding ratio of 89.4 % (Table 1). The genome map of LB-0542 with the contigs, GC content and GC skewness is depicted in Fig. 1. The evolutionary relatedness of the strain LB-0542 based on its genome (Fig. 2) and 16S rRNA gene sequences (Fig. S1), reveals that its closest relative is the *Stutzerimonas marianensis* strain PS1^T^ (GenBank accession no JALGRD000000000) as indicated by a digital DNA-DNA hybridization (dDDH) value of 86.1 % and an average nucleotide identity (ANI) value of 98.3 %. The screening of the genome of LB-0542 via PathogenFinder2 indicates that this strain is non-pathogenic.

**Table 1:** Genome features of the novel *Stutzerimonas marianensis* strain LB-0542.

Feature	*S. marianensis* LB-0542
Genome size	4520,813 bp
Genome coverage	110X
Number of contigs	12
Completeness	99.4 %
Contamination	0.44 %
GC content	63.2 %
Longest contig	1715,002 bp
N50	1079,143 bp
L50	2
Gap ratio	0.0 %
Coding-sequences	4166
rRNA	3
tRNA	56
CRISPR	0
Coding ratio	89.4 %

The results of the genome annotation using the COG (Clusters of Orthologous Genes) database indicate that 71 % of the genes belong to a COG category (Fig. 1 and Table S1). The three dominant COG functional classes, which were divided into 20 COG categories, were recorded as metabolism (34.3 %), cellular process and signalling (22.7 %), and information storage and processing (14 %). Furthermore, the genome annotation via RAST revealed 484 subsystems with 54 % system coverage (Fig. S2). The four dominant subsystem features were recorded as the amino acids and derivatives (382 genes), the carbohydrates (356 genes), the protein metabolism (272 genes) and the cofactors, vitamins, prosthetic groups and pigments (254 genes).

The screening of the genome of strain LB-0542 for the prediction of carbohydrate-active enzymes (CAZymes) via the Protologger [11] revealed the occurrence of 230 genes encoding the CAZymes of four functional classes, glycoside hydrolases [GH (75 genes)], glycosyltransferases [GT (95 genes)], carbohydrate esterases [CE (9 genes)] and carbohydrate-binding modules [CBM (51 genes)] (Table 2). The most abundant families of the GH, GT, CE and CBM were recoded as GH13 (15 genes), GT2 (28 genes), CE11 (4 genes) and CBM50 (17 genes).

**Table 2:** Carbohydrate-active enzymes (CAZymes) in the genome of the novel *Stutzerimonas marianensis* strain LB-0542.

CAZyme class	CAZyme family (number of genes)
Carbohydrate-binding modules (CBM)	CBM2 (2), CBM5 (4), CBM12 (1), CBM13 (2), CBM20 (3), CBM32 (1), CBM41 (9), CBM48 (8), CBM50 (17), CBM51 (2), CBM56 (1), CBM57 (1)
Carbohydrate esterases (CE)	CE4 (1), CE11 (4), CE12 (1), CE14 (2), CE16 (1)
Glycoside hydrolases (GH)	GH0 (11), GH1 (2), GH3 (1), GH5 (5), GH6 (4), GH13 (15), GH16 (1), GH17 (2), GH19 (5), GH23 (9), GH24 (1), GH28 (2), GH37 (1), GH39 (2), GH43 (2), GH65 (2), GH71 (1), GH73 (2), GH77 (1), GH101 (1), GH103 (3), GH114 (1), GH135(1)
Glycosyltransferases (GT)	GT0 (5), GT1 (9), GT2 (28), GT4 (27), GT5 (2), GT9 (3), GT13 (2), GT19 (1), GT20 (1), GT22 (1), GT26 (4), GT28 (5), GT30 (1), GT35 (1), GT51 (4), GT104 (1)

**Fig. 3:**
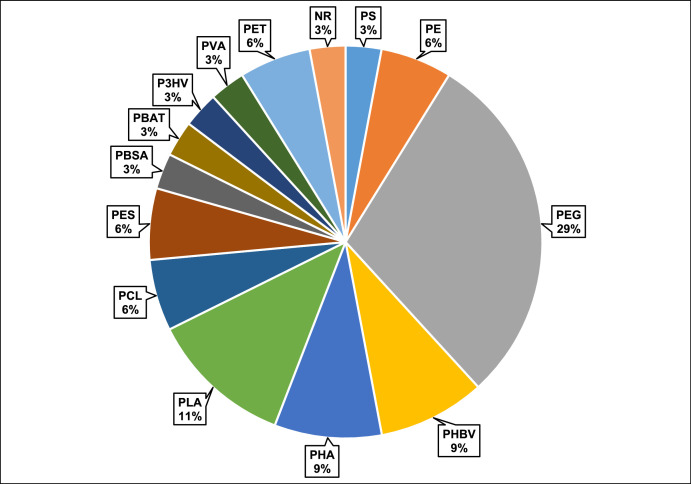
Distribution of plastic-degrading genes detected in the genome of the novel *S. marianensis* strain LB-0542. PEG: polyethylene glycol; PLA: polylactic acid; PHBV: poly(3-hydroxybutyrate-co-3-hydroxyvalerate); PHA: polyhydroxyalkanoates; PE: polyethylene; PCL: polycaprolactone; PES: polyethersulfone; PET: polyethylene terephthalate; PS: polystyrene; PBSA: polybutylene succinate; PBAT: poly(butylene adipate-co-terephthalate; P3HV: poly(3-hydroxyvalerate); PVA: polyvinyl alcohol; NR: natural rubber.

The functional annotation of the genome of strain LB-0542 via the plastic biodegradation database (PlasticDB) [12] predicts the presence of 34 plastic-degrading genes, which can catalyse the degradation process of diverse plastic types, polyethylene glycol [PEG (29 %)], polylactic acid [PLA (11 %)], poly(3-hydroxybutyrate-co-3-hydroxyvalerate) [PHBV (9 %)], polyhydroxyalkanoates [PHA (9 %)], polyethylene [PE (6 %)], polycaprolactone [PCL (6 %)], polyethersulfone [PES (6 %)], polyethylene terephthalate [PET (6 %)], poly(butylene adipate-co-terephthalate [PBAT (3 %)], polystyrene [PS (3 %)], polybutylene succinate [PBSA (3 %)], poly(3-hydroxyvalerate) [P3HV (3 %)], polyvinyl alcohol [PVA (3 %)] and natural rubber [NR (3 %)] (Fig. 3 and Table S2).

**Fig. 4:**
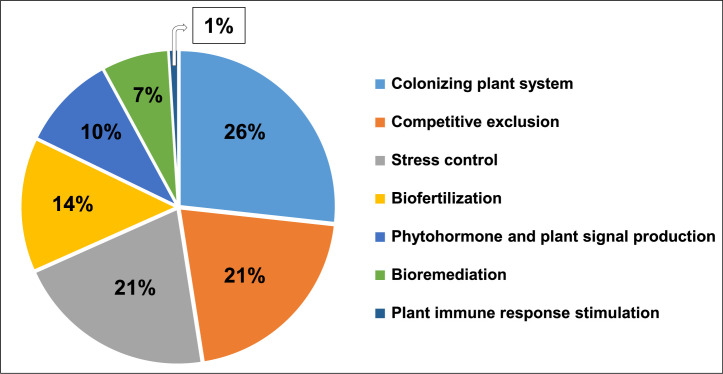
Distribution of plant growth-promoting traits (PGPT)-related genes in the genome of the novel *S. marianensis* strain LB-0542.

Additional genome screening of strain LB-0542 via the Plant Growth-Promoting Traits Prediction (PGPT-Pred) tool [13] revealed the prevalence of 3175 PGPT-related genes (Table S3). These PGPT genes were associated with the colonizing plant system (26 %), competitive exclusion [a bacterial species engaged in competition with other bacterial species for nutrients and mucosal adhesion sites (21 %)], stress control (21 %), biofertilization (14 %), phytohormone and plant signal production (10 %), bioremediation (7 %) and plant immune response stimulation (1 %) (Fig. 4).

## 4 Experimental Design, Materials and Methods

### 4.1 Isolation of the strain LB-0542

The strain LB-0542 was isolated from decomposing *Sargassum* biomass (aka *Sargassum* waste) using tenfold serial dilutions (in sterile seawater) and spread plating on mineral agar (w/v) (0.02 % MgSO_4_, 0.002 % CaCl_2_, 0.1 % KH_2_PO_4_, 0.1 % K_2_HPO_4_, 2 % NaCl, 0.1 % NH_4_NO_3_ and 1.5 % agar; pH 7.5) supplemented with 0.5 % (w/v) carboxymethyl cellulose (CMC) (Sigma Chemicals, Milwaukee, WI) as reported previously [14]. The *Sargassum* waste, which was composed of two holopelagic species, *S. fluitans* and *S. natans*, was collected from Long Beach (13.06°N, 59.49°W), Barbados.

### 4.2 DNA extraction and genome sequencing

The strain LB-0542 was cultivated in tryptic soy broth (BD DIFCO™, Franklin Lakes, NJ) for 48 h at 30 °C. The cells were collected by centrifugation (12,000 g for 10 min at 4 °C) and used for the DNA extraction using InstaGene™ matrix (Bio-Rad Laboratories, Canada). The purification of the extracted DNA was performed with ethanol precipitation [15] and resuspension of the purified DNA in elution buffer (IBI Scientific, Peosta, IA). The quality and quantity of the purified DNA were assessed using the NanoDrop Lite Spectrophotometer and the Qubit 4 Fluorometer (Thermo Fisher Scientific, Waltham, MA), respectively. The whole-genome sequencing was performed via the Illumina NextSeq2000 platform with the paired-end (2 × 150 bp) library preparation protocol using Illumina DNA Prep Kit [16]. The raw reads quality was assessed via FastQC (ver 0.12.1), followed by trimming and filtering of the raw reads using Trimmomatic (ver 0.39).

### 4.3 Genome assembly and annotation

The genome assembly of the strain LB-0542 was conducted via the SPAdes Genome Assembler (ver 3.15.5) with a k-mer sequence of 21,33,55,77,99 for the reduction of mis-assemblies and the removal of low-coverage contigs. Small size contigs of <500 bp were removed. The resulting assembly was checked for completeness and contamination via the CheckM (ver 1.0.18) program available in the DDBJ Fast Annotation and Submission Tool (DFAST) [9] and the ContEst16S (Contamination Estimator by 16S) tool [10]. The assembled genome was annotated using the DFAST pipeline with default settings [9]. A circular genome map was created using the EzBioCloud pipeline [17]. The functional annotations with reference to the clusters of orthologous genes (COG), subsystem features, carbohydrate-active enzymes (CAZymes), plastic degradation and plant-growth promoting traits were performed using the eggNOG mapper (ver. 4.1) [18], Rapid Annotation System Technology (RAST) [19], Protologger (ver 2.0) (confirmed by the three integrated tools, HMMER, DIAMOND and dbCAN-Sub) [11], PlasticDB [12] and PGPT-Pred (blastp+hmmer approach) [13] tools with default settings, respectively. The pathogenic capability of LB-0542 was determined using the PathogenFinder2.0 (ver 0.5.0) (https://genepi.food.dtu.dk/pathogenfinder).

### 4.4 Phylogenomics of LB-0542

The genome-based evolutionary relatedness of the strain LB-0542 was determined via the Type (Strain) Genome Server (TYGS) using the Genome BLAST Distance Phylogeny (GBDP) approach [20]. The genome-based taxonomy of the strain LB-0542 was inferred from the TYGS using two different bioinformatic approaches. In the first approach, the intergenomic relatedness was assessed via a comparative analysis between the genome of LB-0542 and the available type strain genomes in TYGS database. In the second approach, a comparative analysis was performed between the 16S rRNA gene sequence of the strain LB-0542, which was curated from the whole-genome sequence, and the type strains available in the TYGS database. The digital DNA-DNA hybridization (dDDH) values between the genome sequence of the strain LB-0542 and its nearest type strain relatives were determined in TYGS using the formula *d_4_* [20]. The ANI values were determined using the FastANI tool (ver 0.1.3) available in DFAST [9].

## Limitations

None.

## Ethics Statement

The authors have read and followed the ethical requirements for publication in Data in Brief and confirm that the current work does not involve human subjects, animal experiments, or any data collected from social media platforms.

## CRediT Author Statement

**Bidyut R. Mohapatra:** Conceptualization, Methodology, Formal analysis, Writing– original draft, Writing – review & editing, Supervising, Funding acquisition; **Linel S. Moralez:** Writing– original draft, Writing – review & editing, Methodology, Investigation, Data curation; **Kiya E. James:** Writing– original draft, Writing – review & editing, Methodology; Data curation.

## Data Availability

GenBank-National Center for Biotechnology InformationGenome sequence of a novel Stutzerimonas marianensis strain LB-0542 isolated from decomposing Sargassum (Original data).

## Appendix Supplementary materials

Image, application 1
